# Winners take it all: turning cell competition into a therapeutic ally

**DOI:** 10.1186/s12964-025-02611-3

**Published:** 2026-01-07

**Authors:** Tereza Fantová, Maxima Warmuzová, Sandra Charvátová, Kateřina Stošková, Tomáš Jelínek, Michal Šimíček, Roman Hájek, Juli R. Bagó

**Affiliations:** 1 Department of Hematooncology, Faculty of Medicine, University of Ostrava, Ostrava, Czech Republic; 2 Department of Hematooncology, University Hospital Ostrava, Ostrava, Czech Republic

**Keywords:** Cell competition, Winner cells, Loser cells, Super-Competition, Cancer, Regenerative medicine, Aging, Senescence, Pluripotent stem cells, EDAC, Therapy, Apoptosis.

## Abstract

Recent discoveries in the field of cell competition have brought this phenomenon into the spotlight due to its potential to inspire novel therapeutic approaches for previously incurable diseases.

Cell competition, a process observed across multicellular organisms, acts as a natural quality control mechanism in which unfit, abnormal, or malignant cells are actively eliminated by neighboring, healthy-fit cells. However, the mechanisms and outcomes of cell competition are highly context-dependent, influenced by the environment, developmental stage, and specific tissue involved. Emerging research also highlights a darker aspect where cancer cells can hijack cell competition to their advantage, promoting tumor growth and progression. A deeper understanding of cell competition opens up a wide range of therapeutic possibilities for diseases that currently lack effective treatments. Given its specificity and context-dependence, we categorize these therapeutic opportunities based on the physiological settings in which cell competition occurs.

## Background

Cell competition is not a new concept, it was first described by Morata and Ripoll in 1975 [1]. However, recent discoveries have brought renewed attention to this phenomenon across diverse biological contexts, including cancer, tissue regeneration, aging, and senescence. These discoveries have reignited interest in the field, positioning it as a rapidly evolving area with significant therapeutic potential.

The earliest insights into cell competition arose from studies in *Drosophila melanogaster*, where cells harboring mutations in ribosomal proteins and referred to as *Minute* mutants, were shown to be actively eliminated when surrounded by wild-type cells (WT). This interaction, termed competition between “winner” (WT) and “loser” (mutant) cells, results in loser cells death due to activation of Jun N-terminal kinase (JNK)-dependent apoptosis [2].

Over the past two decades, it has become clear that cell competition is evolutionarily conserved across both invertebrates and vertebrates [3–5]. It occurs not only during development but also in adult tissues such as heart, ovarian epithelium, gut, skin and brain [6–9]. In the course of development, cell competition was elegantly demonstrated by Cristina Claveria et al. and Margarida Sancho et al. [10, 11], who showed that mouse embryonic stem cells with higher *Myc* levels and referred as fit, eliminate neighboring cells with lower *Myc* levels and referred as unfit. This *Myc*-driven competition serves as a quality control mechanism in the embryo, ensuring that vulnerable, mispatterned, or abnormal cells with lower expression of *Myc* are removed from the forming epiblast. Similarly, in adult tissues, cell competition plays a critical role in maintaining homeostasis and tissue integrity. In this context, cell competition can be viewed as a primitive surveillance mechanism that identifies and eliminates viable but suboptimal cells, thus preserving overall tissue function.

However, cell competition is not exclusively beneficial. It can also manifest in a phenomenon known as super-competition, in which cells that acquire a competitive advantage, often through oncogenic mutations, expand and eliminate otherwise healthy, optimal cells. These super-competitor cells ultimately dominate the tissue compartment [12]. This process is particularly relevant in cancer, where super-competitor tumor cells outcompete both less-fit tumor cells (intra-tumor competition) and normal tissue cells [13]. Therefore, cell competition can act as a double-edged sword, with outcomes highly dependent on the physiological or pathological context in which it occurs.

**Fig. 1:**
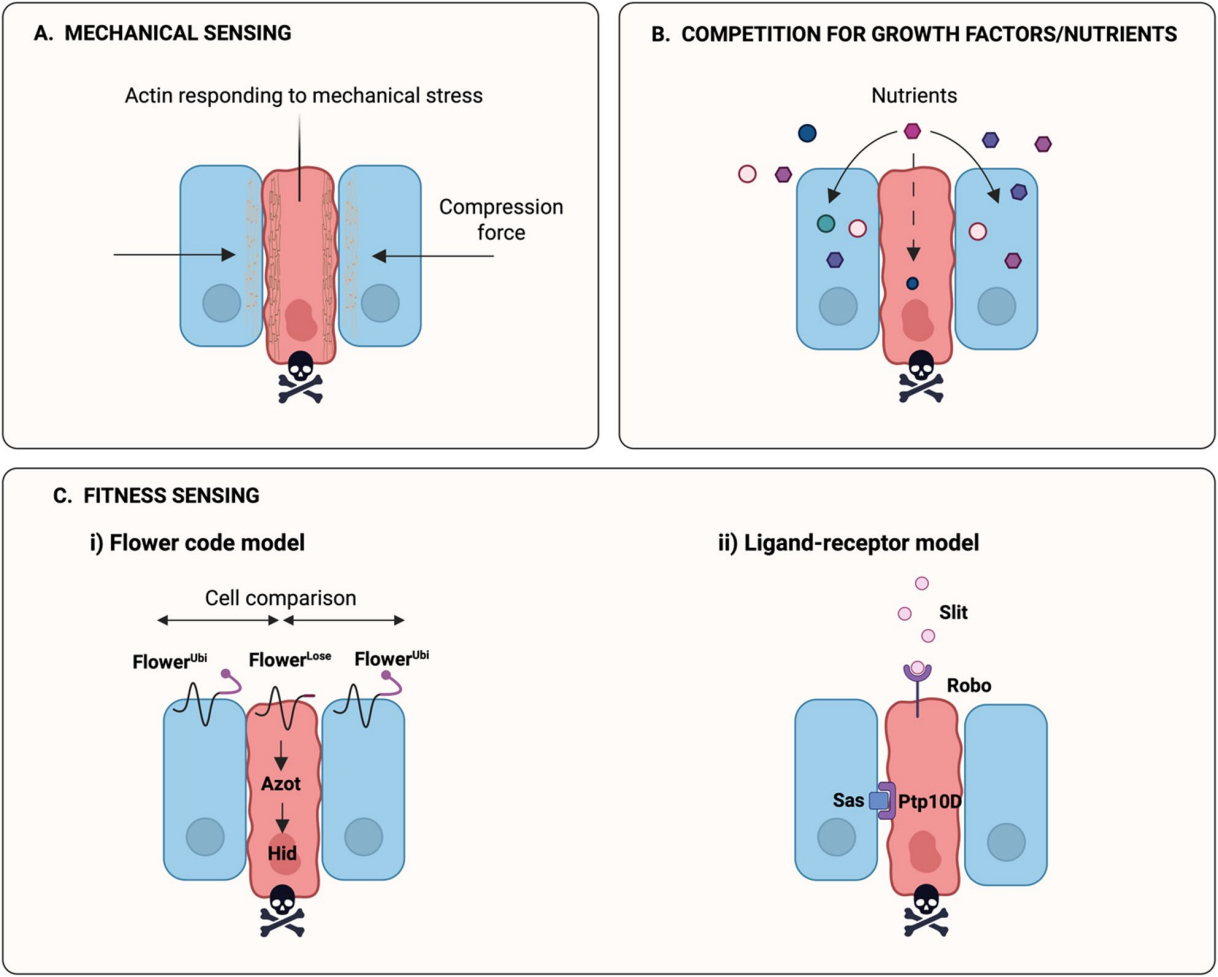
Diagram depicting the distinct mechanisms by which cells sense and eliminate their less fit neighbors through cell competition. **A** Mechanical sensing. Compression induces actin rearrangement that leads to extrusion and death of mechanically stressed cells. **B** Competition for growth factors and nutrients. Cells compete for access to limiting nutrients, less competitive cells undergo cell death. **C** Fitness sensing: (**i**) Flower code model. Cells express fitness markers (Flower isoforms) and differences on their expression triggers apoptosis in less fit cells via *azot* and *hid *(**ii**) Ligand–receptor model. Interactions between ligands (Slit, Sas) and receptors (Robo, Ptp10D) mediate elimination of unfit cells. Loser cells are shown in blue, and winner cells in red. Created in https://BioRender.com

To date, three primary modes of cell competition have been described. The most recently characterized is mechanical competition (Fig. 1A), which occurs when physical forces within a tissue lead to the elimination of less fit cells through actin rearrangement. For instance, Levayer et al. [14] demonstrated in *Drosophila* that increased local cell density can lead to the extrusion and apoptosis of loser cells through delamination and caspase activation. Another well-established mechanism is competition for survival factors (Fig. 1B), in which cells compete for limited survival signals, such as cytokines or growth factors. In the mammalian thymus, for instance, progenitor cells compete for interleukin-7. Older, less responsive cells are eliminated in favor of younger, more responsive ones [15]. The third mode described is fitness sensing-based competition (Fig. 1C), which includes two distinct models. The first one, flower fitness sensing model (Fig. 1C i), involves isoforms of the transmembrane protein Flower. Specific isoforms mark cells as losers, targeting them for elimination [16]. It was first described in *Drosophila* and is conserved in mice and humans. The second model, known as ligand receptor interaction model (Fig. 1C ii), involves winner cells expressing surface ligands (e.g., Sas in *Drosophila*) that interact with receptors on neighboring loser cells, triggering JNK signaling and apoptosis [17].

**Fig. 2:**
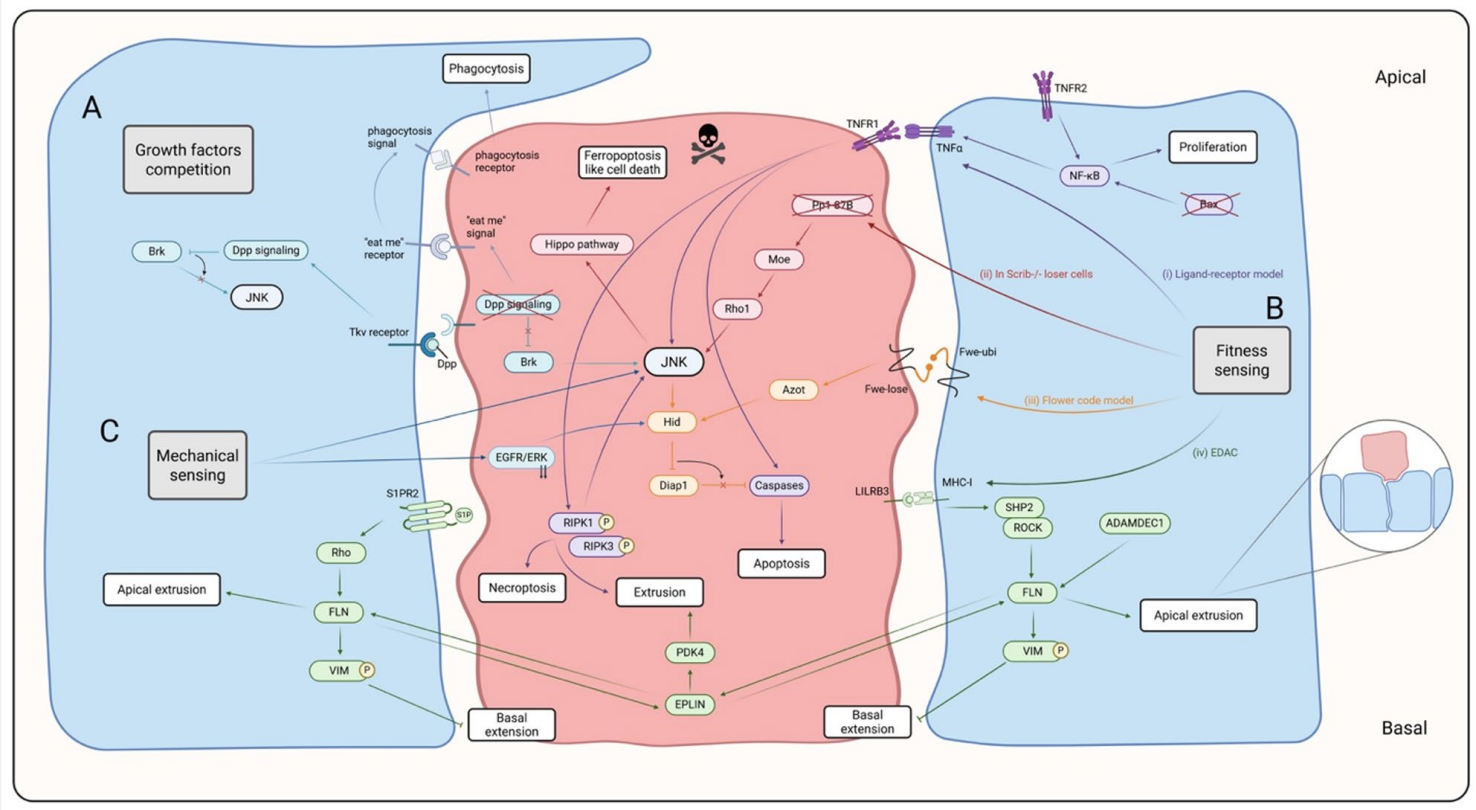
Cell competition modes and their mechanism of elimination. **A** Cell competition for growth factors and nutrients model and loser cells elimination by JNK-dependent apoptosis and phagocytosis. In *Drosphila*, cells with reduced *dMyc* expression (loser cells) receive insufficient levels of the trophic factor Decapentaplegic (Dpp), which is required for growth and survival. Dpp deprivation induces the expression of Brinker (Brk), leading to activation of the JNK apoptotic pathway. In this model, loser-cell elimination can also occur through phagocytosis: compromised Dpp signaling induces “eat-me” signals that promote engulfment by neighboring non-compromised cells [2]. **B** (i) Cell competition fitness sensing ligand-receptor model in mammalian hair follicle stem cells (HFSCs) and loser cells elimination by apoptosis. Loss of the pro-apoptotic protein Bax in winner HFSCs elevates nuclear factor-kappa B (NF-κB)-dependent tumor necrosis factor α (TNFα) expression and its accumulation at the membrane. Binding of TNFα to TNFR1 on neighboring loser cells activates caspase-dependent apoptosis [23]. Cell competition fitness sensing ligand-receptor model in mammalian Madin-Darby canine kidney (MDCK) cells and loser cells elimination by necroptosis and extrusion. Upstream inducer of necroptosis still not defined but might include FASL, TRAIL, and Toll-like receptor which triggers necroptosis and extrusion through receptor-interacting protein kinase 1 (RIPK1) and receptor-interacting protein kinase 3 (RIPK3) [18]. (ii) Cell competition fitness sensing model in *Drosophila* and loser cells elimination by ferroptosis like cell death. Loss of Pp1-87B in loser cells activates JNK signaling through the Moesin–Rho1 axis, which in turn activates Hippo signaling and induces ferroptosis-like cell death [22]. (iii) Cell competition fitness sensing flower code model in *Drosophila* and loser cells elimination by apoptosis. Loser cells expressing transmembrane specific isoform (*fwe*^*lose*^*)* activate the expression of *azot* that in turn activates *Hid*-dependent apoptosis [24]. (iv) Cell competition cell fitness sensing model and loser cells elimination by EDAC-mediated extrusion. Normal epithelial cells expressing leukocyte immunoglobulin-like receptor B3 (LILRB3) recognize transformed loser cells displaying major histocompatibility complex class I (MHC class I). This interaction promotes filamin (FLN) and vimentin (VIM) accumulation at winner–loser boundaries. Exogenous sphingosine-1-phosphate (S1P) also produce FLN and VIM accumulation through Rho/Rhok pathway. In loser cells, epithelial protein lost in neoplasm (EPLIN) and plectin are mobilized, driving cytoskeletal remodeling required for apical extrusion [25]. **C** Cell competition mechanical sensing model and loser cells elimination by apoptosis and extrusion. In the *Drosophila* epithelium, mechanical compression imposed by neighboring over-proliferating cells reduces EGFR/ERK survival signaling in loser cells. This downregulation increases the pro-apoptotic factor Hid, activates caspases, and culminates in the apical extrusion of loser cells during tissue crowding [14]. Loser cells are shown in blue, and winner cells in red. Created in https://BioRender.com

The most common mechanism by which non-optimal loser cells are eliminated is cell death, primarily through necroptosis [18] and the JNK-dependent apoptosis [19] (Fig. 2). In some instances, autophagic cell death has also been observed in loser cells [6]. Additionally, active engulfment of apoptotic loser cells by neighboring winner cells has been reported [20] (Fig. 2). Another well documented elimination route is cell extrusion, especially observed in epithelium (Fig. 2). In this case, loser cells with oncogenic mutations, such as *RasV12* and *Trp53*, are apically extruded by winner WT epithelium cells. The extruded cells encounter a hostile environment that will result in cell death by anoikis or necroptosis. This process is known as epithelial defense against cancer (EDAC) [21]. A recent study identifies a new mechanism for eliminating loser cells through a ferroptosis-like form of cell death, which is triggered by activation of the Hippo signaling pathway (Fig. 2) [22].

In a nutshell, cell competition is an inherent quality control mechanism in multicellular organism which is context-dependent, with the corresponding outcome depending on the environment, development stage and specific tissue. In this review, we aim to highlight the therapeutic opportunities that recent discoveries in cell competition offer, categorizing them according to the physiological context in which they occur.

### Cell competition in cancer

The relationship between cell competition and cancer is complex, ranging from tumor suppression to facilitation of malignancy. While some competitive interactions contribute to maintaining tissue homeostasis and preventing oncogenic transformation, others are hijacked by malignant cells to promote tumor growth and resistance to therapy. One of the well-studied tumor-suppressive manifestations of cell competition, previously described in this review, is EDAC. EDAC represents a natural defense ability of epithelial cells, enabling them to recognize and eliminate potentially harmful, transformed neighbors via apical extrusion [21, 26]. This competitive elimination helps maintain tissue homeostasis and reduces the risk of tumor formation. For instance, in vivo studies have demonstrated the clearance of RasV12-expressing cells from murine intestinal and pancreatic epithelium via this mechanism [21, 27].

However, normal WT cells are not always the winners in competitive interactions. Transformed cells can evolve strategies to escape tissue surveillance and even subvert the cell competition machinery. This shift is often referred to as super-competition, a phenomenon where oncogenic cells hijack cell competition mechanism to their advantage [28]. For instance, cells overexpressing *Myc*, a well-known oncogene, can outcompete their WT counterparts, leading to clonal expansion and potentially initiating tumorigenesis [29]. Understanding when and how malignant cells subvert cell competition mechanisms opens up new possibilities for targeted therapies. Therapeutically targeting the competitive advantage given to tumoral cells could reduce cancer aggressiveness and prevent clonal dominance by highly competitive subpopulations. Another promising approach could be enhancing the competitive fitness of WT cells to restrain cancer progression [30].

For instance, targeting the tumor suppressor *p53* could inhibit super-competitor status in tumor cells. *p53* is frequently mutated and inactivated in the majority of malignant tumors [31]. It has long been viewed as a highly appealing target for anti-cancer drug development. However, for many years, *p53* was considered “undruggable,” and progress in creating effective *p53*-targeted therapies remained limited [32]. Studies have shown that activation of *p53* in normal cells can enhance their ability to eliminate neighboring transformed cells through cell competition mechanisms. For instance, normal cells with intact *p53* signaling have been shown to outcompete and eliminate less-fit or oncogene expressing neighbors cells through a fitness-sensing competition mechanism, leading to their senescence or apoptosis [33]. In epithelial systems, mechanical cues such as crowding and compression can also activate *p53*, sensitizing cells to apoptotic elimination under competitive stress [34]. These findings support a role for *p53* in promoting early tumor suppression by strengthening the ability of normal cells to sense and remove potentially malignant cells from the tissue.

**Fig. 3:**
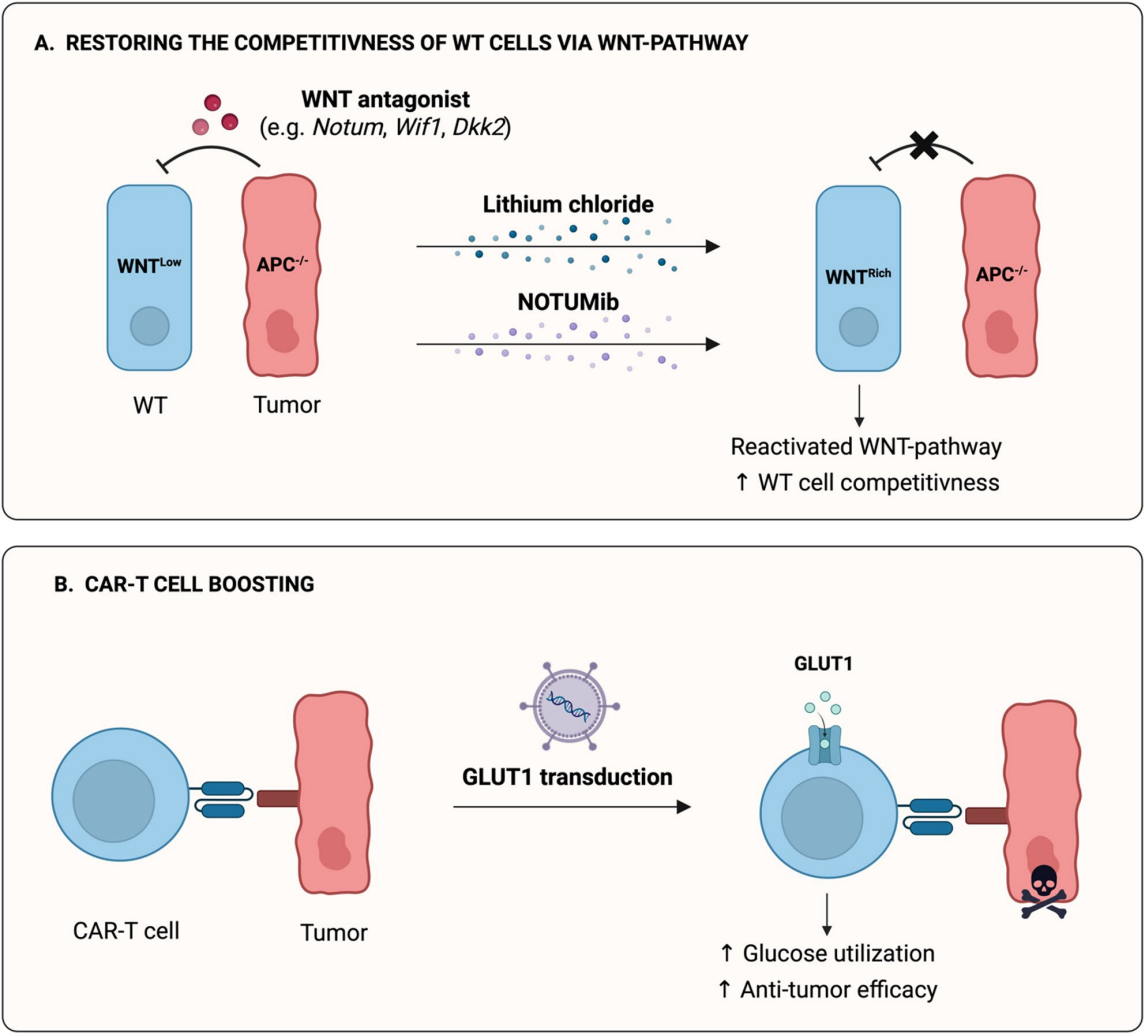
Strategies to modulate cell competition in the tumor microenvironment. **A** Restoring the competitiveness of WT cells by reactivating the WNT signaling pathway by treatment with agents such as lithium chloride or NOTUMib, thereby increasing the competitive fitness of WT cells against tumor cells harboring APC mutations. **B** Enhancing anti-tumor efficacy of CAR-T cells through increased cellular competitiveness with the overexpression of glucose transporter GLUT1 which boosts glucose uptake and metabolic activity of CAR-T cells, improving their ability to eliminate tumor cells. Created in https://BioRender.com

A similar principle applies to the loss of adenomatous polyposis coli (APC), a tumor suppressor gene whose inactivation is a hallmark of colorectal cancer [35]. APC functions as a negative regulator of the Wnt signaling pathway. Its loss leads to aberrant activation of Wnt target genes that promote uncontrolled proliferation and impaired differentiation of intestinal stem cells. Neerven et al. demonstrated that APC-mutant intestinal stem cells gain a significant competitive advantage over their WT counterparts by actively secreting Wnt signaling antagonists (NOTUM) which inhibits proliferation of adjacent cells and triggers their differentiation. Interestingly, pharmacological downstream activation of Wnt signaling by lithium chloride or inhibition of the Wnt antagonist NOTUM restored the fitness of WT cells and reduced mutant clone dominance (Fig. 3A) [35, 36]. Beyond Wnt signaling, downstream effectors such as NF-κB also modulate competitive outcomes. Pharmacological inhibition of NF-κB restored lost EDAC function in WT cell, reducing invasiveness in mouse intestinal epithelium and organoid models [37]. These findings underscore how transformed cells exploit cell competition to promote invasion but also reveal avenues for therapeutic reactivation of defense mechanisms.

Another interesting study, by Hosseini et al. [38], demonstrated how metformin, a widely used antidiabetic drug, reduced the clonal fitness of DNMT3A-mutant hematopoietic stem cells in vitro and in transplantation models. This resulted in decreased self-renewal capacity and clonal dominance of leukemic cells, highlighting a clinically relevant example of pharmacological modulation of cell competition.

Cell competition is also gaining recognition in the field of cancer immunotherapy. Immune cell subsets, such as CD8 + T cells and natural killer (NK) cells, compete for access to cytokines and niche signals. Song et al.. demonstrated that tumor-associated NK cells can outcompete CD8 + T cells for interleukin-2 and interferon-alpha, thereby reducing antitumor immunity and limiting the efficacy of immune checkpoint therapies [39]. This also suggests that modern immunotherapy could leverage the principles of cell competition. For instance, engineered immune cells such as chimeric antigen receptor (CAR)-T and CAR-NK cells can be seen as “external competitors” that selectively eliminate tumor cells. A recent study by Shi et al. demonstrated that CAR-T cells engineered to overexpress the glucose transporter GLUT1 exhibited enhanced metabolic fitness and increased anti-tumor activity in both solid tumors and leukemia (Fig. 3B) [40].

Building on the concept of leveraging cell competition for cancer treatment, our team explored the potential of a cell-based antitumor approach [41]. In this context, we identified urine progenitor cells (UPCs) as a promising candidate due to their high intrinsic cellular fitness and non-invasive accessibility from voided urine. Remarkably, co-culturing UPCs with various tumor cell lines led to tumor cells death in a cell-cell contact-dependent manner. These findings highlight the potential of UPCs as a novel cell source for cancer therapy and demonstrate how cell competition can be harnessed as a therapeutic strategy.

The study of these competitive dynamics has been demonstrated in in vitro and in vivo models of cell competition. Approaches such as organoid cultures [42], co-culture systems [43], fluorescent barcoding [44], and CRISPR-based screening techniques [45] enable the identification of genes and pathways involved in cell competition. For instance, García’s group used intestinal organoids to show that transformed cells can eliminate WT neighbours via JNK-dependent apoptosis. Inhibition of this pathway preserved WT cells and reduced tumor expansion [42]. More recently, the same group demonstrated that altering cell competition in murine organoids and microtissues affects liver metastasis formation [46].

Taken together, these studies demonstrate that cell competition is not merely a passive process, but rather a targetable phenomenon by pharmacological and cellular interventions which can limit clonal expansion, suppress malignancy, and enhance therapeutic efficacy. A deeper understanding of these dynamics may lead to more precise interventions that harness the intrinsic competitive behaviours of cells to prevent or treat cancer.

### Cell competition in regenerative medicine

Regenerative medicine has emerged as a promising strategy to replace, rejuvenate, and repair damaged tissues and organs, offering new therapeutic avenues for previously untreatable diseases [47]. This field utilizes external interventions such as gene and cell therapies, as well as the body’s own innate healing mechanisms. In both approaches, cell competition can be leveraged to enhance regenerative outcomes, either by promoting the expansion of fitter cells within damaged tissues or by selecting and transplanting fit cells to improve engraftment, homing, and propagation. Notably, in both scenarios, the fitter cells may actively eliminate damaged or unfit cells, contributing to complete tissue regeneration.

**Fig. 4:**
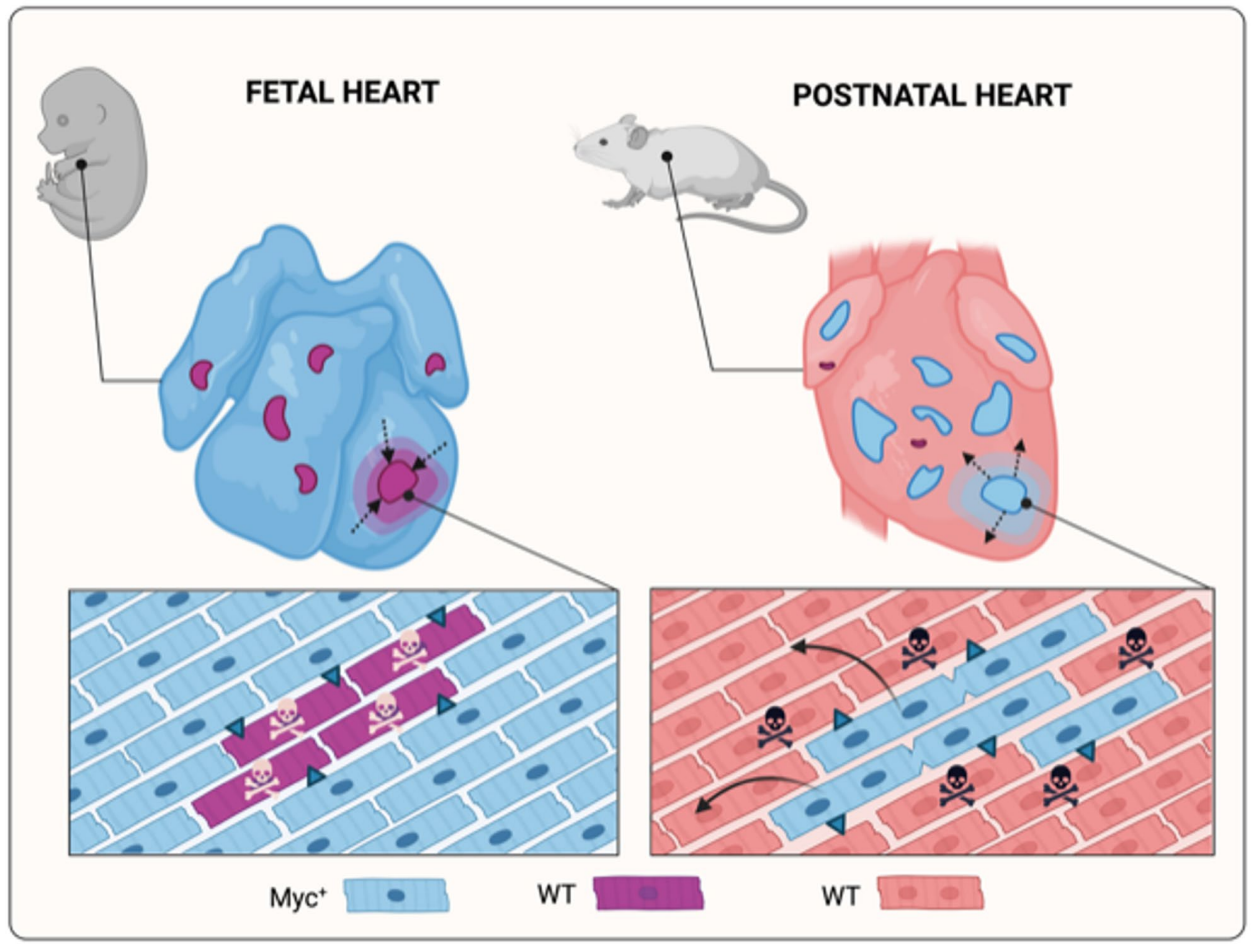
Schematic representation of cell competition in heart. Cell competition of cardiomyocytes during development and cell competition of postnatal cardiomyocytes. Arrows indicating direction of cell replacement. Blue cells showing winner cells (Mycn + in development and Myc^+^ in postnatal) and red-purple loser cells (wild type-WT). Created in https://BioRender.com

This phenomenon of cell replacement has been observed in the transplantation of fit embryonic hepatocytes into adult liver tissue. The transplanted hepatocytes successfully engrafted and, due to their higher fitness, outcompeted and replaced endogenous hepatocytes, leading to their elimination [48]. Similar findings have been reported in other organs, such as the heart. Cristina Villa del Campo et al. [6] demonstrated that cell competition can be induced in both embryonic and adult mouse hearts through mosaic overexpression of *Myc* in cardiomyocytes. During embryonic development, *Myc*-overexpressing (fit) cardiomyocytes actively eliminated neighboring cells with lower *Myc* expression via short-range signaling or direct cell-cell contact, triggering apoptosis without inducing hyperplasia (Fig. 4). A more recent study [49] indicates that, among the three members of the *Myc* family of transcription factors (*Myc*, *Mycn*, and *Mycl*), only *Mycn* is required for heart development. However, *Myc* can compensate for the loss of *Mycn* during cardiomyocyte development, and cardiomyocyte competition depends on the levels of both *Mycn* and *Myc*.

In adult hearts, a similar pattern of cell competition was observed. However, the elimination of less fit cells occurred through autophagic cell death rather than apoptosis (Fig. 4). Another key difference in adult hearts was the increased proliferation of the winner cardiomyocytes, likely compensating for the loss of loser cells, as no hypertrophy was detected. Importantly, despite a 20-fold overexpression of *Myc*, tissue homeostasis remained uncompromised. This finding opens the door to future regenerative medicine strategies that could harness cell competition in the heart to promote tissue renewal and repair.

It is important to note that the activation of cell competition does not always lead to tissue homeostasis. For example, Marianna Yusupova et al. [23] demonstrated that inducing cell competition in hair follicle stem cells (HFSCs) by depleting the pro-apoptotic *Bax* gene resulted in tissue hypertrophy. Remarkably, their study revealed a fitness-sensing mechanism based on a ligand-receptor interaction model in mouse HFSCs. Specifically, *Bax*-knockdown HFSCs eliminated non-engineered neighboring HFSCs by expressing TNF-α, which bound to TNF-α receptor 1 (TNFR1) on the loser cells, triggering their apoptosis. Although this cell competition promoted hair production and tissue regeneration following epidermal injury, it also led to hypertrophy and abnormal tissue growth in grafts derived from *Bax*-deficient HFSCs. These findings highlight the need for precise control when leveraging cell competition in regenerative medicine, as unintended outcomes may compromise therapeutic benefits.

### Cell competition in aging and senescence

Recent advances in diagnostic and medicine allowed to extend life expectancy and quality [50]. Despite this progress, ageing is still one of main risk factors that endanger human health and linked to aged-related diseases such as neurodegeneration [51], musculoskeletal disorders [52], and cardiovascular diseases [53]. Marianna Marques-Reis et al. [54] suggest a model for cell competition in aging, in which young organisms harbor low amount of loser cells or damaged cells. These cells are rapidly and efficiently eliminated by neighboring winning cells. However, as the organism ages, the amount of damaged cells increase due to a higher likelihood of acquiring mutations [55]. This leads to an imbalance in favor of damaged cells, reducing the effectiveness of their elimination by winner cells and thereby accelerating the aging process. Latest studies claim that it can be reversed by promoting the removal of non-optimal loser cells, opening new therapeutic avenues to tackle aging related diseases. Indeed, contemporary literature underscores the potential of cell competition to mitigate neurodegenerative diseases such as Alzheimer’s disease (AD) by promoting the elimination of damaged neurons. While the removal of neurons in the brain may initially seem counterproductive, Coelho et al. [56]. demonstrated that the clearing of damaged neurons enables the formation of new synaptic connections between healthy neurons, thereby restoring proper neural function. In alignment with the amyloid cascade hypothesis, which posits that amyloid-β accumulation in neurons drives neurodegeneration in AD, these researchers developed amyloid-β-transgenic flies. In this model, they observed fitness sensing-based competition: suboptimal neurons expressing amyloid-β displayed the loser isoform of the Flower protein (Flower^LoseB^), while neighboring, healthier neurons expressed the winner isoform (Flower^ubi^). Loser neurons were identified and targeted for apoptosis by their fitter neighbors.

The apoptotic elimination of loser neurons required downstream activation of the gene *azot*, which in turn activated the pro-apoptotic gene *hid*. Inhibition of apoptosis led to worsened locomotor impairments, similar to those observed when *azot* expression was reduced. Remarkably, motor function was restored by introducing an additional copy of *azot* in neurons and removing apoptosis inhibitors. Based on these findings, the authors proposed a therapeutic strategy centered on enhancing cell competition. This could involve promoting apoptosis with Bcl-2 or Bcl-xL inhibitors such as BH3-mimetic drugs [57] or through *azot* overexpression.

A recent preprint from Eduardo Moreno’s team [58] further explored the role of diet in modulating AD progression through cell competition. In this study, the researchers compared flies fed with a yeast-based diet to those on a synthetic diet. They found that the synthetic diet was associated with lower levels of amyloid-β, reduced number of loser neurons, and improved locomotor performance. Conversely, the yeast-based diet resulted in higher amyloid-β, higher number of loser neurons, and a decline in motor function. These results suggest that dietary composition can influence neurodegeneration by modulating cell competition pathways.

Ten years ago, another study lead by Eduardo Moreno [59] emphasized the critical role of cell competition in extending lifespan. Flies lacking the gene *azot* exhibited a significantly shortened lifespan, whereas those carrying three functional copies of the gene experienced a 54% increase in median survival. Additionally, dietary restriction further extends lifespan, particularly in flies with three functional copies of *azot*. Although not explicitly stated in the manuscript, it is reasonable to infer that, beyond dietary restriction, the same therapeutic approach proposed for enhancing cell competition in AD, such as promoting apoptosis with Bcl-2 or Bcl-xL inhibitors, could similarly contribute to lifespan extension.

**Fig. 5:**
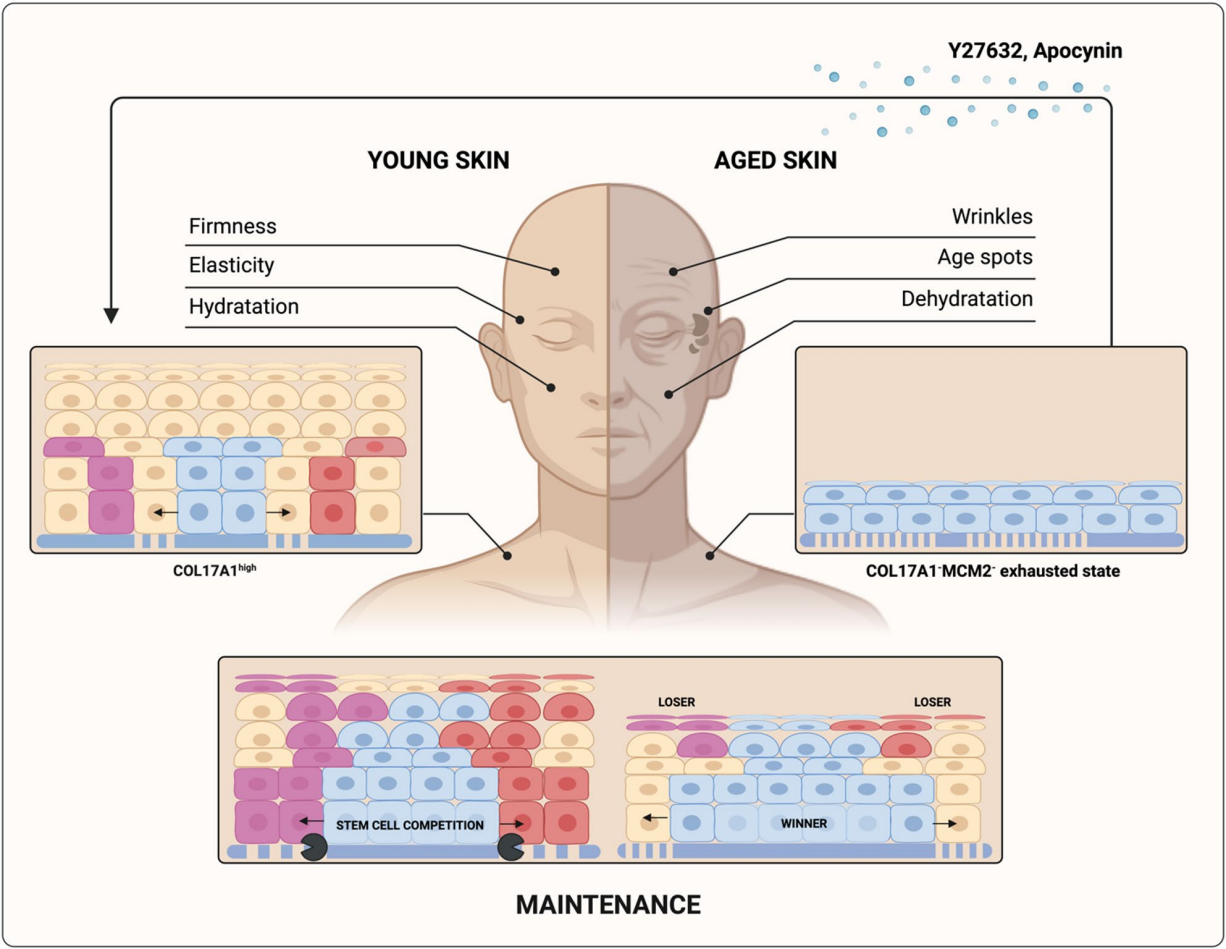
Schematic illustration of epidermal aging driven by COL17A1 expression which mediates cell competition in the epidermis. In young skin, tissue homeostasis is maintained through continuous cell competition, where unfit or stressed keratinocytes with low COL17A1 expression are eliminated by stem cell keratinocytes expressing high levels of COL17A1. As the skin ages, COL17A1 expression in stem cell keratinocytes declines, leading to reduced cell competition and contributing to the aged skin phenotype. To counteract this process, compounds such as Y27632 and apocynin, known inducers of COL17A1 expression in keratinocytes, have been proposed as potential interventions. Winner cells are shown in blue, while loser cells are shown in yellow-red-purple. Created in https://BioRender.com

In a more recent study [60], Liu et al. investigated how epidermal stem cell keratinocytes orchestrate skin homeostasis and aging through cell competition. They discovered that fit epidermal stem cells, characterized by high expression of COL17A1, continuously compete and eliminate unfit or stressed keratinocytes with lower COL17A1 expression (Fig. 5). This form of cell competition appears to be both fitness-sensing and mechanical driven, as loser cells are removed from the basal interfollicular epidermis by delamination. The mechanical force securing fit COL17A1^+^ to the epidermal basement membrane is generated by their parallel symmetric cell division, allowing these cells to spread horizontally along the basement membrane. Contrarily, unfit COL17A1^−^ cells undergo perpendicular division, which limits their retention in the basal layer. Notably, delaminated loser cells in this system are not eliminated by apoptosis. With aging, this competition mechanism declines, leading to an increased number of COL17A1^−^ keratinocytes exhibiting perpendicular cell division within the basement membrane (Fig. 5). This results in skin fragility, atrophy (skin thinning), delay in wound healing and dyspigmentation of the skin [61, 62]. As a potential therapeutic approach, the authors proposed the use of two chemical compounds known for their capacity to induce COL17A1 expression in keratinocytes: Y27632 and apocynin. When applied to skin wounds in mice, these compounds significantly improved healing outcomes, comparable to those observed in mice genetically engineered to overexpress COL17A1 in keratinocytes.

### Cell competition in pluripotent stem cells

Since their discovery by Shinya Yamanaka in 2006 [63], human induced pluripotent stem cells (hiPSCs) have become one of the primary cell culture systems in modern biology. Given their increasing relevance, it is imperative to include in this review recent studies examining cell competition phenomena observed during hiPSCs cultivation. hiPSCs provide an unlimited source of cells and possess the capacity to differentiate into all three germ layers. This makes them invaluable for a wide range of applications, including investigations into stem cell behavior, lineage-specific differentiation for tissue modeling, disease modeling, and as a cellular source for regenerative therapies and cancer treatments [64–66]. In all these applications, maintaining a genetically homogeneous population is critical.

**Fig. 6:**
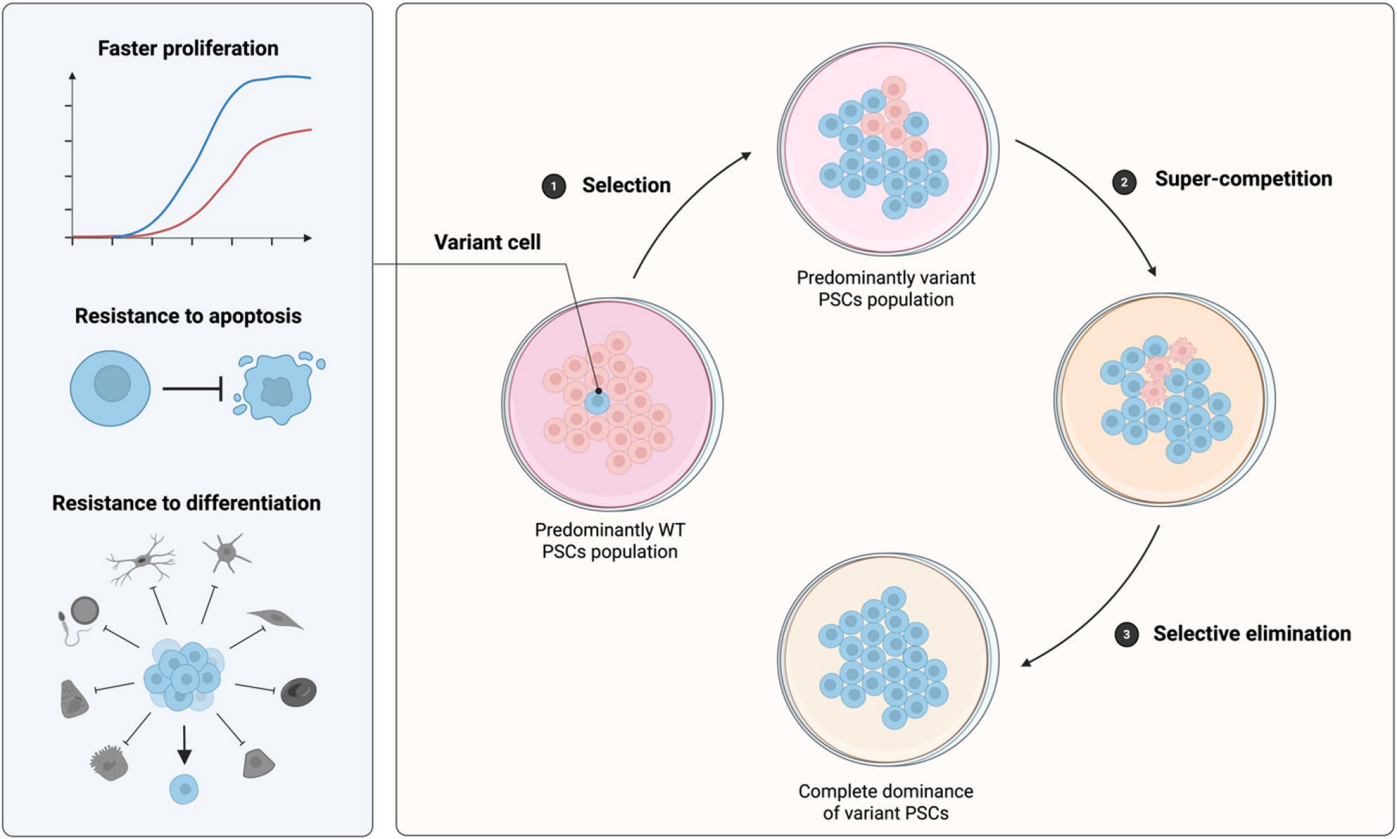
Mechanisms of cell competition in pluripotent stem cells (PSCs) cultivation. Selection: variant PSC with growth advantages (e.g., increased proliferation, resistance to apoptosis or differentiation) progressively overtake WT PSCs in culture. Super-competition: variant PSCs actively induce the elimination of neighboring WT PSCs through mechanical cell competition, ultimately leading to complete dominance within the culture. Created in https://BioRender.com

However, multiple studies have raised concerns about the emergence of subpopulations within hiPSCs cultures that harbor genetic abnormalities [67]. These variant cells often arise during the reprogramming process [68] and exhibit a competitive growth advantage, ultimately overtaking the culture during extended passaging. This advantage is typically associated with enhanced proliferation, resistance to apoptosis, and resistance to differentiation (Fig. 6). Nevertheless, these factors alone do not fully explain the rapid dominance of variant cells in hiPSC cultures [69].

To address this, Price et al. [69] conducted time-lapse microscopy studies on mixed populations of genetically variant hiPSCs, bearing commonly observed mutations, and WT hiPSCs lacking detectable mutations. Their findings revealed super-competition, that was driven by mechanical competition. Variant cells with higher proliferative capacity physically confine the WT cells, leading to the re-localization of the transcriptional coactivator YAP from the nucleus to the cytoplasm in WT cells. This shift ends up triggering the apoptosis in the WT population (Fig. 6). To counteract this effect, the authors proposed the use of YAP nuclear localization enhancers such as the SETD7 methyltransferase inhibitor R-PFI-2 [70]. This treatment effectively blocked super-competition, thereby reducing the complete overtake of the culture by variant hiPSCs.

However, it is important to note that R-PFI-2 does not eliminate the inherent selective advantages of variant hiPSCs, such as faster proliferation and apoptosis resistance. Therefore, to truly minimize their dominance, the use of R-PFI-2 should be combined for instance with small molecules survival enhancers such as CEPT cocktail [71] or culture strategies designed to limit cellular stress [72]. An open question is whether the super-competition phenomenon also occurs in hiPSCs cultured in suspension. Large-scale hiPSC production relies on scalable and efficient systems, such as bioreactor-based expansion, where cells are grown as aggregates in suspension [73]. Therefore, understanding how super-competition behaves in this context is critically important for minimizing the emergence of variant hiPSCs during large-scale manufacturing.

## Conclusions and perspectives

The concept of cell competition has been described as a remnant of social adjustment mechanisms in unicellular organisms, drawing a strong parallel with Darwin’s theory of natural selection [74]. In multicellular systems, this process provides an extra layer of defense, contributing to the maintenance of tissue homeostasis and integrity. Recent discoveries in the field have offered new insights into the role of cell competition in a wide range of biological processes that can be leveraged for novel therapeutic strategies that we outlined in this review.

**Fig. 7:**
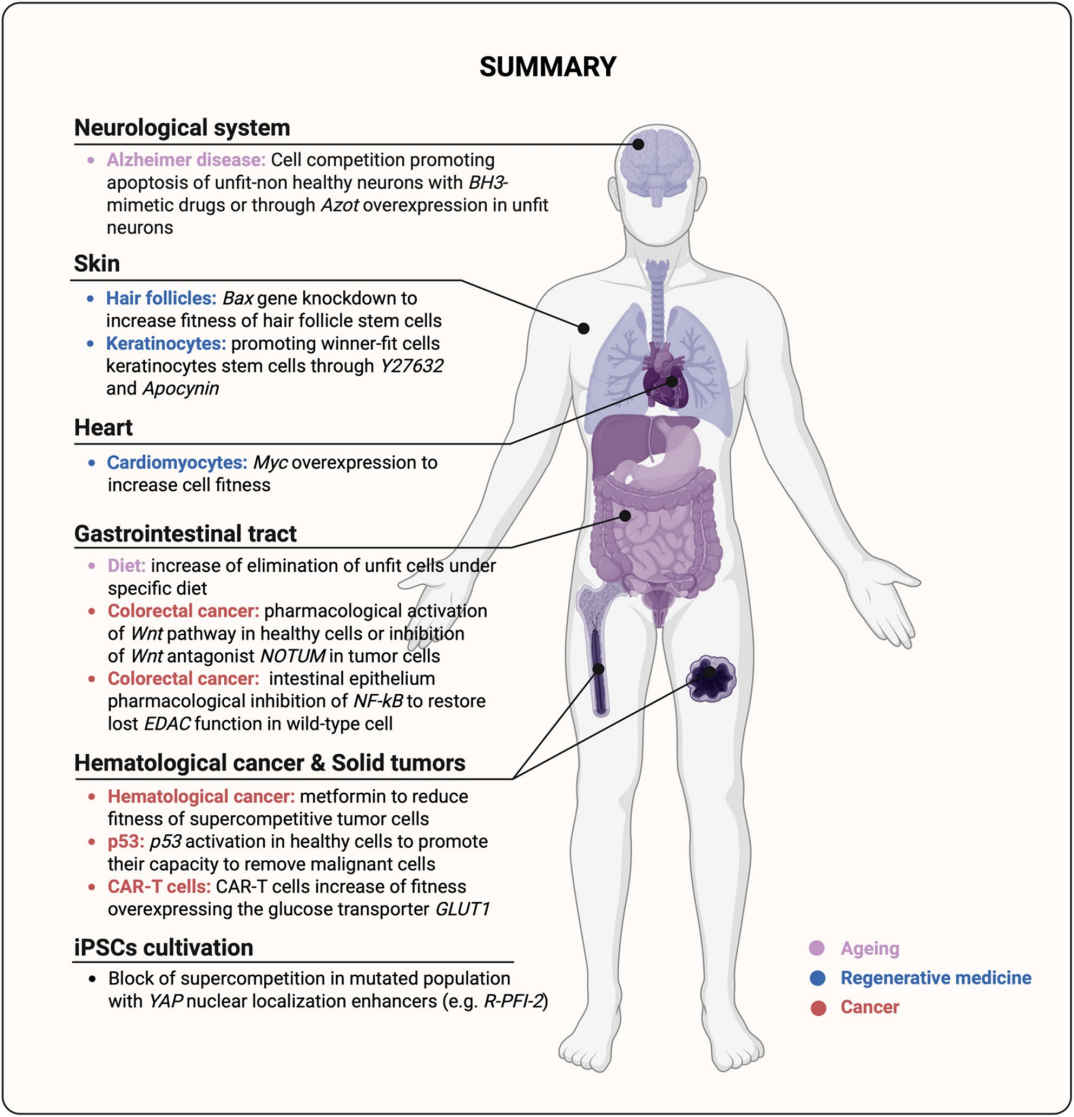
Schematic illustration of therapeutic strategies discussed in this review that leverage cell competition across various tissues and pathophysiological contexts. Created with https://BioRender.com

In oncology, the dual nature of cell competition, acting both as a safeguard against transformation and as a mechanism that can be hijacked by malignant cells, adds further complexity. While mechanisms such as EDAC and super-competition have been well characterized, the next frontier lies in harnessing these dynamics for clinical benefit. Strategies such as *p53* activation, Wnt pathway modulation, or NF-κB inhibition have shown promise in enhancing the competitive fitness of WT cells, restoring their capacity to eliminate mutant competitors [33–37] (Fig. 7 and Table 1). This raises the exciting possibility that selective pressure within tissues could be tipped in favour of normal cells, thereby containing or even reversing early tumorigenesis. In this context, therapeutic approaches may need to consider not only how to reinforce healthy competitors or suppress malignant ones, but also how to strategically combine both effects. Reshaping this competitive landscape in such a way could open new avenues for delaying therapy resistance and minimizing the risk of relapse.

**Table 1:** Overview of druggable targets, system-specific indicators, and corresponding interventions in which cell competition is leverage

System	Application / Indication	Druggable target	Intervention	Ref.
Neurological	Alzheimer’s disease	BCL-2/BCL-xL anti-apoptotic proteins; Azot pathway	BH3-mimetic drugs (BCL-2/BCL-xL inhibitors); Azot overexpression to promote apoptosis of unfit neurons	[56–58]
Cardiovascular	Cardiac disease	Myc in cardiomyocytes	Myc overexpression to enhance fitness of cardiomyocytes	[6]
Gastrointestinal	Alzheimer’s disease and lifespan	Metabolic pathways modulated by diet (amyloid-β reduction)	Dietary modulation (synthetic diet, dietary restriction) to enhance elimination of loser cells	[58, 59]
	Colorectal cancer	Wnt signaling pathway in healthy intestinal cells	Pharmacologic downstream activation of Wnt signaling (e.g. lithium chloride) in wild-type cells	[35, 36]
	Colorectal cancer	NOTUM (secreted Wnt antagonist) in APC-mutant tumor cells	Pharmacologic inhibition of NOTUM (e.g. NOTUM inhibitors) in tumor cells	[35, 36]
	Colorectal cancer	NF-κB signaling (NF-κB-MMP21 axis) in intestinal epithelium to restore EDAC function	Pharmacologic inhibition of NF-κB (blocking NF-κB-MMP21 pathway) to restore EDAC	[37]
Hematopoetic	Leukemia	Metabolic pathways of Dnmt3a-mutant hematopoietic stem cells	Treatment with metformin to reduce clonal fitness of Dnmt3a-mutant cells	[38]
	Malignant tumors	p53 pathway in healthy hematopoietic and epithelial cells	Pharmacologic activation of p53 in healthy cells to strengthen competitive elimination of transformed cells	[31–34]
	Malignant tumors	GLUT1 glucose transporter in CAR-T cells	Genetic overexpression of GLUT1 to increase metabolic fitness of CAR-T cells	[40]
Skin	Hair regeneration	Bax in hair follicle stem cells	Bax gene knock-down/depletion in HFSCs to create fitter winner cells	[23]
	Skin regeneration	COL17A1 in epidermal stem cell keratinocytes	Chemical induction of COL17A1 expression using Y27632 and apocynin	[60–62]
iPSC	iPSC cultivation	YAP nuclear localization / survival pathways in hiPSCs	Use of YAP nuclear localization enhancers (e.g. R-PFI-2) and culture optimization (e.g. CEPT cocktail, low-stress culture) to block supercompetition of variant hiPSCs	[69–72]

Moreover, cell competition is also relevant in hematologic malignancies and cancer immunotherapy, where agents like metformin can suppress pre-leukemic stem cells, such as those harbouring DNMT3A mutations [38] (Fig. 7 and Table 1). In parallel, immunotherapeutic approaches, including CAR-T and CAR-NK cells, can be viewed as engineered “external competitors” designed to specifically eliminate tumor cells. Might enhancing the metabolic or signaling fitness of these immune effectors, as seen with GLUT1-overexpressing CAR-T cells [40] (Fig. 7 and Table 1), represent the next leap in cancer immunotherapy?

Beyond cancer, in the field of aging and age-related diseases, Eduardo Moreno’s team has explored how promoting apoptosis in unfit neurons, specifically those burdened with amyloid-β accumulation, can alleviate neurodegenerative phenotypes. By using BH3-mimetic drugs or overexpressing the *azot* gene [56] (Fig. 7 and Table 1), they observed significant improvement, underscoring the therapeutic potential of selectively eliminating dysfunctional cells. In contrast, Liu et al. [60] proposed another approach. Rather than removing unfit cells, they enhanced the functionality of fit-winner cells to maintain skin homeostasis. Through the application of Y27632 and apocynin (Fig. 7 and Table 1), they induced the expression of COL17A1 in keratinocytes, reinforcing cellular fitness and competition in the skin. It would be particularly interesting to assess whether combining both strategies, apoptosis of unfit cells alongside promotion of fit cells, could produce synergistic effects in enhancing tissue health and delaying degenerative processes.

Although not explicitly tied to cell competition, Alejandro Ocampo et al. [75] made a groundbreaking contribution to aging research by demonstrating that partial reprogramming with Yamanaka factors (Oct4, Sox2, Klf4, and c-Myc) can epigenetically reset aging markers. In both mouse and human cells, this intervention corrected key hallmarks of aging, including DNA damage, senescence, and epigenetic dysregulation. Notably, in a premature aging mouse model, cyclic induction of these factors extended lifespan and restored tissue homeostasis. Despite the therapeutic promise, there are well-documented risks associated with in vivo expression of Yamanaka factors, particularly the potential for teratoma formation and cancer [76]. However, this approach opens the door to cellular rejuvenation as a form of age intervention therapy. A deeper understanding of how partial reprogramming affects cell fitness dynamics could reveal a hidden layer of cell competition, where reprogrammed, rejuvenated cells might outcompete damaged or aged counterparts, restoring tissue integrity in a controlled fashion.

Overall, these studies spark the imagination with visions of the long-sought “fountain of youth”, yet they also remind us of the immense complexity of multicellular organisms. Any successful therapeutic approach will require precise and context-dependent strategies to harness the beneficial aspects of cell competition without unleashing their potential hazards.

 Such undesired side effects have been also observed in the field of regenerative medicine. For instance, increasing cell competition in hair follicles by promoting winner cells with the depletion of *Bax* gene [23] (Fig. 7 and Table 1) led to increased hair production and enhance wound healing capacity, but also resulted in hypertrophy and abnormal tissue growth. In contrast, work by Cristina Villa del Campo et al. [6]. demonstrated that the promotion of winner cells in the heart, through *Myc* overexpression (Fig. 7 and Table 1), could enhance cardiac homeostasis without inducing hypertrophy, intensifying the high specificity and context-dependence of cell competition.

In summary, the therapeutic promise of cell competition lies in its versatility but so do its risks. As seen in regenerative models, tipping the balance in favour of fit winner cells can drive repair and rejuvenation, yet may also provoke abnormal growth or tissue dysregulation if not carefully controlled. Tissue-specific and context-dependent effects demand a nuanced understanding and tailored strategies. Moving forward, a deeper mechanistic understanding, combined with precision tools to steer competitive dynamics, will be essential to safely harness cell competition for regenerative, anti-aging, and anti-cancer therapies.

## Data Availability

No datasets were generated or analysed during the current study.

## Abbreviations

APC: Adenomatous polyposis coli
AD: Alzheimer’s disease
Brk: Brinker
CAR: Chimeric antigen receptor
CRISPR: Clustered regularly interspaced short palindromic repeats
Dpp: Decapentaplegic
EDAC: Epithelial defence against cancer
EPLIN: Epithelial protein lost in neoplasm
FLN: Filamin
HFSCs: Hair follicle stem cells
hiPSCs: Human induced pluripotent stem cells
JNK: Jun N-terminal kinase
LILRB3: Leukocyte immunoglobulin-like receptor B3
MDCK: Madin-Darby canine kidney
MHC class I: Major histocompatibility complex class I
NF-kB: Nuclear factor-kappa B
NK: Natural killer
RIPK1: Receptor-interacting protein kinase 1
RIPK3: Receptor-interacting protein kinase 3
PSCs: Pluripotent stem cells
S1P: Sphingosine-1-phosphate
TNFα: Tumor necrosis factor α
TNFR1: TNF-α receptor 1
UPCs: Urine progenitor cells
VIM: Vimentin
WT: Wild-type
